# Impact of Global DNA Methylation in Treatment Outcome of Colorectal Cancer Patients

**DOI:** 10.3389/fphar.2018.01173

**Published:** 2018-10-18

**Authors:** Mariam A. Fouad, Salem E. Salem, Marwa M. Hussein, Abdel Rahman N. Zekri, Hafez F. Hafez, Eman D. El Desouky, Samia A. Shouman

**Affiliations:** ^1^Pharmacology Unit, Department of Cancer Biology, National Cancer Institute, Cairo University, Cairo, Egypt; ^2^Department of Medical Oncology, National Cancer Institute, Cairo University, Cairo, Egypt; ^3^Virology and Immunology Unit, Department of Cancer Biology, National Cancer Institute, Cairo University, Cairo, Egypt; ^4^Department of Biostatistics and Epidemiology, National Cancer Institute, Cairo University, Cairo, Egypt

**Keywords:** colorectal cancer, fluoropyrimidine therapy, 5 methylated cytosine, DNA methyl transferase, survival analysis

## Abstract

**Background:** Global DNA methylation has an impact in cancer pathogenesis and progression. This study aimed at investigating the impact of global DNA methylation in treatment outcome of Colorectal Cancer (CRC).

**Patients and Methods:** Global DNA methylation was measured by LC/MS/MS in peripheral blood leucocytes of 102, 48, and 32 Egyptian CRC patients at baseline and after 3 and 6 months of Fluoropyrimidine (FP) therapy respectively, in addition to 32 normal healthy matched in age and sex. The genetic expressions of DNA methyl transferases (DNMTs) were determined and correlated with patients‘ survival using univariate and multivariate methods of analyses.

**Results:** Egyptian CRC patients had significant global hypomethylation of 5mC level and 5mC % with overexpression of DNMT3A and DNMT3B. Significant higher 5mC levels were shown in patients > 45 years, male gender, T2 tumors, stage II, negative lymph nodes, and absence of metastasis. FP therapy significantly reduced DNA methylation particularly in the subgroups of patients with high DNA methylation level at baseline and good prognostic features. After 3 years of follow up, patients with 5mC % > 8.02% had significant poor overall survival (OS) while, significant better event-free survival (EFS) was found in patients with 5mC level > 0.55. High initial CEA level and presence of metastasis were significantly associated with hazards of disease progression and death.

**Conclusion:** Global DNA methylation has a significant impact on the treatment outcome and survival of Egyptian CRC patients treated with FP- based therapy.

## Background

Colorectal cancer incidence and mortality rates are still rising rapidly. It is expected that by 2030, global burden will increase by 60% with 2.2 million new incidences and 1.1 million deaths especially in developing countries rather than developed countries (Arnold et al., 2017).

Epigenetic alterations are contributed in the pathogenesis, molecular heterogeneity, and progression of CRC disease (Moore et al., 2013). The best characterized epigenetic modification is DNA methylation which is a covalent addition of a methyl group (CH3–), obtained from S- adenosyl methionine to the 5 position of a cytosine base within CG dinucleotides by DNMTs to form 5-methylcytosine (5mC).

DNMT1, DNMT3A, and DNMT3B have been identified as DNA methylation functional enzymes in eukaryotic cells. DNMT1 is often referred to as the maintenance methyltransferase which is responsible for maintaining pre-existing methylation patterns during DNA replication (Leonhardt et al., 1992). Whereas DNMT3A and DNMT3B are the de novo methylation enzymes, and their importance is correlated with the embryogenesis and pathogenesis of cancer cell (Yang et al., 2011). The levels of DNMT3B and DNMT3A are often increased in various cancer tissues and cell lines (Okano et al., 1998). They cause hypermethylation of promoter CpG-rich regions in a variety of malignancies (Yun et al., 2012) and repress transcription in either methylation-dependent manner (Linhart et al., 2007; Nosho et al., 2009) or through their histone deacetylase activity (Fuks et al., 2001). Moreover, DNMT3B has been shown to play a role in leukemia development and maintenance of Leukemia stem cell function (Schulze et al., 2016). Contrary to their canonical *de novo* methylation role, a series of experiments using DNMT genetic knockout cell lines suggest DNMT1 and DNMT3B cooperate to maintain methylation in human cancers (Rhee et al., 2002).

Global hypomethylation of the entire genome and hypermethylation of specific CpG sites become one of the characteristics of colon cancer (Subramaniam et al., 2014). The hypoxic microenvironment in solid tumors is known to contribute to inappropriate silencing and re-awakening of genes involved in cancer progression through local epigenetic alterations (Shahrzad et al., 2007). Global DNA hypomethylation in peripheral blood leukocytes was found to be a potential biomarker for CRC risk (Nan et al., 2013) and breast cancer risk (Kuchiba et al., 2014). The stability of DNA methylation as molecular marker and its effect on genes expression facilitates its clinical use in early cancer detection, and make it an attractive target to predict the treatment outcome and patients‘ response to therapy (Ogino et al., 2008).

Fluoropyrimidine based therapy is the standard treatment for all stages of CRC. The anticancer efficacy of FP- therapy is highly dependent on multiple factors as intracellular folate concentration, DNMTs expression, methylation background of colonic mucosa and silencing of critical FP metabolizing genes (Farias et al., 2015). The development of chemoresistance to FP- therapy was reported to be associated with the methylation of DNA of mismatch repair gene and incidence of colorectal recurrence (Sinicrope et al., 2011). Tumors methylation phenotypes were correlated with patients‘ response to chemotherapy and some studies have identified global DNA methylation as an independent marker for survival and response to FP based therapy in CRC patients (Iacopetta, et al., 2008; Baharudin et al., 2017).

Therefore, this study was designed to investigate the impact of global DNA methylation machinery on the clinical outcome of Egyptian CRC patients treated with FP- based therapy.

## Patients and Methods

### Samples Collection and Lymphocytic Cell Pellet Preparation

This is a prospective study of the effect of global DNA methylation on the treatment outcome of CRC patients. Patients included in this study were with confirmed diagnosis of CRC who were enrolled to the National Cancer Institute, Cairo University during the period from February 2014 to December 2014. Whole blood samples were collected from CRC patients at baseline, then after 3 and 6 months of FP based therapy respectively. In addition to 32 whole blood samples were collected from healthy controls that were matched in age and sex. Mononucleated leukocyte cell pellets were isolated by hemolysin buffer (8.46 g ammonium chloride, 1 g potassium bicarbonate and 1 g ethylene diamine disodium salt dissolved in 1 L and the pH = 7–7.2).

### Global DNA Extraction and DNA Hydrolysis

The whole genomic DNA was extracted from leukocytes by blood DNA preparation and purification Kit (Jena Bioscience, PP 205S, Germany). DNA hydrolysis was performed with triple enzymatic hydrolysis method as described by Crain (1990). One μg of extracted genomic DNA was denatured by heating at 100°C for 3 min then chilled on ice. A mixture of ammonium acetate (0.1 M, pH 5.3) and 2 units of nuclease was incubated with the denatured DNA sample at 45°C for 2 h. Ammonium bicarbonate(1 M), venom phosphodiesterase I (0.002 unit) and alkaline phosphatase (0.5 unit) were subsequently added to the mixture and incubation was continued at 37°C for 2 h.

### Global DNA Methylation

Global genomic DNA methylation level was determined using (LC/MS/MS) adopting the method determined by Ma et al. (2009). The LC-MS-MS system consists of Agilent 1200 HPLC system (Agilent Technologies, Santa Clara, CA, United States), with a quaternary gradient pump (Agilent 1260 infinity), connected with an online vacuum degasser, column oven and autosampler (Agilent 1260 infinity), coupled to a ABSCIEX Q TRAP 3200 mass spectrometer (ABSCIEX, Applied Biosystem, Germany) equipped with an electrospray ionization (ESI) interface. Data acquisition was performed with analyst 4.0 software (ABSCIEX). The quantification of the global contents of 5mC and 2deoxycytosine (2dC) were performed with multiple reaction monitoring (MRM) (AB Sciex software, Germany) where argon gas collision-induced dissociation of 2dC into the ion transitions pair of m/z 228.1 (molecular ion)/112.2 (fragment ion) and dissociation of 5mC into 242.1 (molecular ion)/126.3 (fragment ion). Calibration curves for 5mC and 2dC had increasing amounts from 0 to 100 ng/ml were constructed. The level of 5mC was determined in ng/DNA and 5mC % was calculated by dividing 5mC level to the total cytosine pool as demonstrated in the formula [5mC/(2dC + 5mC)] × 100.

### RNA Extraction and DNMT3A and DNMT3B Genes Expression

Total RNA was extracted from the lymphocytic cell pellet with total RNA purification kit (Direct-zol RNA Kit, Zymo Research, R2050, Germany). Complementary DNA synthesis was performed according to the manufacturer’s instructions using Revert Aid First Strand cDNA synthesis kit (Thermo Fisher). Real-time PCR was conducted by Applied Biosystems syber green PCR master mix. Reverse and forward sequences of primer genes of DNMT3A, DNMT3B, and β-actin were purchased from Invitrogen by (Thermo Fisher Scientific, United Kingdom. DNMT3A forward primer was 5′TTATGGGGATCCTGGAGCGG3′ and the reverse primer was 3′TCTCAGCCGTATCACACTCG5, DNMT3B forward primer was 5′CTACACACAGTCCCTGAGACG3′ and the reverse primer was 3′GTGTCGTCTGTGAGGTCGAT5′. Housekeeping gene β-actin forward primer was 5′CCAGAGCAAGAGAGGTATCC3′ and the reverse primer was 3′CTGTGGTGGTGAAGCTGTAG5′. Cycle threshold (CT) values for genes were normalized to the CT values of β-actin (Δ Ct) and gene relative expression was calculated as (2^-ΔCt^).

### Statistical Analysis

IBM SPSS statistical package version 24 was used in data manipulation. Numeric data explored for normality using Kolmogorov–Smirnov test and Shapiro–Wilk test. Categorical data were expressed as numbers and percentages while numerical data were summarized as medians and interquartile ranges (IQR). Patients were stratified into subgroups according to their clinicopathological and molecular features. More than two subgroups of patients were tested for significance with Kruksil–Wallas test and the pairwise comparison was done by Mann–Whitney. The effect of FP based therapy on global DNA methylation over time (3 and 6 months) was tested by Friedman test. Pairwise comparison of the effect of time was tested by Wilcoxon matched. The correlation between methylated cytosines content and the expression of DNMT genes was tested by Spearman correlation analysis. After 3 years of follow up, OS and EFS of patients were tested by Kaplan and Meier procedure. OS was calculated from the date of diagnosis to the date of death from any cause. Living patients or patients lost to follow-up were censored on the last known alive date. EFS was calculated from the date of resection or neoadjuvant therapy to the date of recurrence, progression or death, which occurred first. EFS for patients who neither progressed, relapsed, nor died, was censored at last assessment prior to loss of follow-up. Significant clinicopathological and molecular variables on patients‘ survivals were tested for their hazardous effects on either death or progression using multivariate COX proportion hazard model. The hazard ratio indicates the risk of death during the OS time or the risk of progression during the EFS time, in one group of patients who showed the high level of a variable (table variables) compared with the other group which showed the low level of a variable (reference variables). All P-values are two-sided and P-values < 0.05 were considered significant.

The research protocol was approval by the Institutional Human Research Ethics Committee of NCI, Egypt, number 00004025, and conducted in accordance with the Declaration of Helsinki. A written informed consent was taken from the participated patients and the full clinico- pathological information was recorded from the patients’ files.

## Results

### Patients’ Clinicopathological Characters and Treatment Protocol

**Supplementary Table S1** illustrates clinicalo- pathological characteristics and the type of treatment received of 102 Egyptian naïve CRC patients. The mean age ± SD of the patients was 45 ± 13.7 while their median was 46 years ranging from 19 to 72 years. The mean ± Standard deviation of age of healthy controls was 39 ± 13.8 and the male: female ratio = 1: 1.6, *P*-value = 0.16. Nearly half of the patients (46%) were ≤45 years with slight male predominance and rectal cancer was diagnosed in 39.2% the patients. Most of the cases had good performance status (ECOG I) while the rest had PS II or III. High initial CEA and CA19.9 levels were recorded in 41.7 and 20.8% of patients, respectively. It was found that about two thirds (63.72%) of the patients had their primary tumor located in the left side while the right side location was encountered in 29.41% and rectal cancer represented 39.2% of all CRC patients. Adenocarcinoma (69.60%), grade II (80.39%) and T3 tumors (63.72%) were the most common pathological subtypes. Thirty-one patients presented with metastatic disease where the liver was the most frequent site followed by the peritoneum then the lung. The treatment protocol was mainly FP based therapy.

### CRC Patients Have Global DNA Hypomethylation With Overexpression of DNMTs 3A and B

**FIGURE 1:**
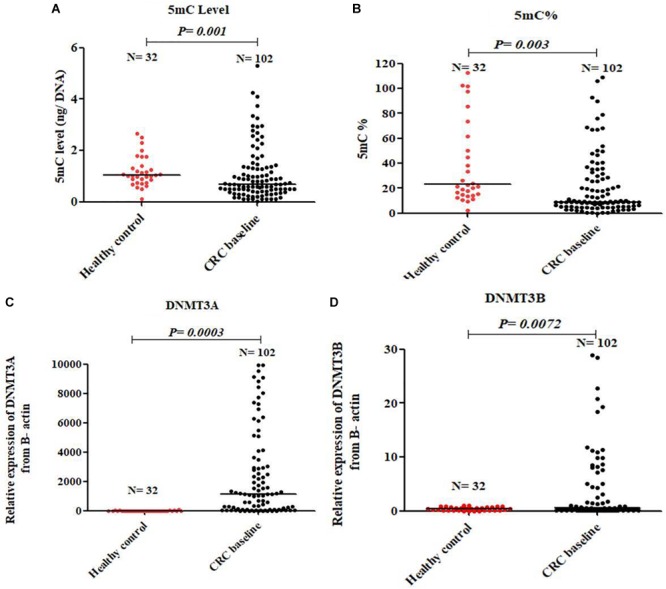
Global DNA methylation in healthy control and baseline CRC patients. 5mC level **(A)**, 5mC % **(B)**, DNMT3A **(C)**, and DNMT3B **(D)**.

At baseline, CRC patients showed global DNA hypomethylation in the form of significant decrease in both 5mC level and 5mC % compared to normal healthy control **Figures 1A,B**. The median level of 5mC and 5mC % for 102 patients at baseline was 0.55 and 8.02 respectively. On the other hand, a significant overexpression of DNMT3A and DNMT3B was observed in CRC patients compared to healthy control **Figures 1C,D**. An inverse correlation of borderline significance was shown between 5mC level and DNMT3A expression (*r* = -0.21 and *P*-value = 0.09), **Supplementary Figure S1**.

### FP Therapy Reduced Significantly 5mC Level, 5mC %, and DNMTs Genes Expression

**FIGURE 2:**
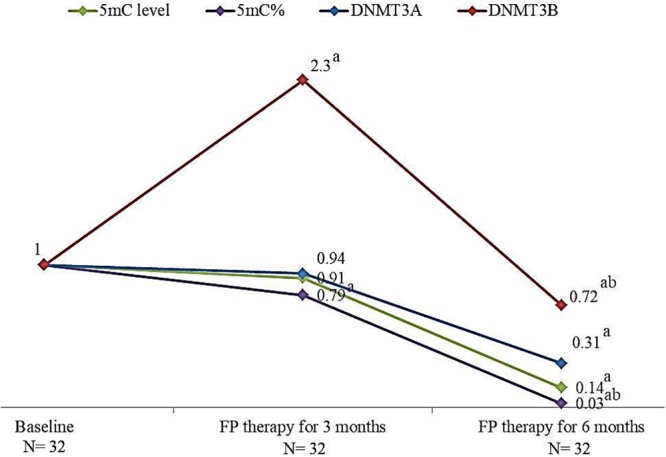
Change in the median levels of 5mC, 5mC %, DNMT3A and DNMT3B genes after 3 and 6 months of FP therapy normalized to their baseline levels. a is a significant difference of CRC patients treated with FP for 3 and 6 months were compared with their baseline level, *P*-value < 0.05. b is a significant difference when CRC patients treated with FP for 6 months were compared with their level after 3 months of treatment, *P*-value < 0.05.

The effect of FP therapy on global DNA methylation and DNMTs gene expression is shown in **Figure 2**. After 6 months of FP therapy, 5mC level, 5mC %, DNMT3A, and DNMT3B were significantly reduced than their corresponding baseline levels. After 3 months of FP therapy, DNMT3B was significantly overexpressed by 2.3 folds while 5mC % was significantly down-expressed to 0.79 folds compared to baseline. 5mC % and DNMT3B gene were significantly reduced after 6 months of FP- therapy compared to the level at the third month.

### 5mC Level and 5mC % in Subgroups of CRC Patients

Using the medians for subgroup analysis of our CRC patients at baseline, Mann–Whitney test of significance showed that patients who were >45 years, males, right-sided location, T2 tumors, grade III, negative lymph nodes, absence of metastasis and stage II have higher 5mC levels. FP therapy significantly reduced the global methylation in patients who had the higher level of 5mC.

Using the pairwise comparison with Wilcoxon matched test of significance, FP therapy significantly reduced 5mC level in CRC patients with T2 tumors after 3 months. While after 6 months of FP therapy, 5mC significantly reduced in patients > 45 years, males, adenocarcinoma subtype, T2, and T3 tumors. The level of 5mC was significantly reduced after 6 months relative to 3 months of FP therapy in patients with T3 tumors and adenocarcinoma pathological subtype **Table 1**.

**Table 1:** Global 5mC levels in subgroups of patients at baseline and after treatment with FP therapy.

Variables		5mC level (ng/ DNA) in CRC patients						*P*-Value
		At baseline		After 3 months		After 6 months		
				of FP therapy		of FP therapy		
			
		*N*	Median (IQR)	*N*	Median (IQR)	*N*	Median (IQR)	
Age	≤45	47	0.59 (0.30–0.60)	23	0.55 (0.14–1.26)	15	0.53 (0.24–0.85)	0.368
	>45	55	0.70^∗^ (0.68–2.09)	25	0.46 (0.28–0.71)	17	0.20**^a^** (0.17–0.35)	***0.011***
	***P*- value**		***0.001***		0.835		0.054	
Sex	Female	47	0.45 (0.38–0.70)	20	0.43 (0.15–1.26)	13	0.29 (0.21–0.45)	0.692
	Male	55	0.86^∗^ (0.79–1.83)	28	0.55 (0.28–0.67)	19	0.20**^a^** (0.17–0.45)	***0.007***
	***P*- value**		***0.029***		0.694		0.669	
CEA level	Normal	46	0.59 (0.44–0.74)	5	0.63 (0.43–0.71)	4	0.19 (0.16–0.25)	0.438
	High	33	0.58 (0.44–0.73)	13	0.51 (0.15–1.26)	8	0.25 (0.20–0.45)	0.053
	***P*- Value**		0.930		0.416		0.237	
CA19.9 level	Normal	57	0.59 (0.44–0.74)	2	0.61 (0.58–0.63)	1	0.17 (0.17–0.17)	NA
	High	15	0.52(0.39–0.65)	17	0.55 (0.28–1.26)	11	0.28 (0.20–0.43)	0.368
	***P*- Value**		0.959		0.449		0.500	
Tumor location	Right colon	30	0.73^∗^ (0.62–1.04)	15	0.67 (0.17–3.45)	16	0.26 (0.20–0.44)	0.097
	Left colon	25	0.47 (0.35–0.59)	15	0.58 (0.14–0.69)	6	0.20 (0.17–0.28)	0.135
	Rectum	40	0.67 (0.5–0.84)	18	0.46 (0.38–1.26)	10	0.30 (0.15–1.08)	0.189
	***P*- value**		***0.006***		0.295		0.441	
Pathology	Adenocarcinoma	66	0.53 (0.43–0.71)	32	0.53 (0.28–0.99)	22	0.21**^ab^** (0.16–0.53)	***0.030***
	Mucinous	34	0.46 (0.50–0.84)	16	0.29 (0.15–0.81)	10	0.30 (0.28–0.43)	0.368
	***P*- value**		0.06		0.342		0.315	
Tumor grade	II	82	0.58 (0.44–0.73)	34	0.46 (0.17–0.67)	24	0.30 (0.20–0.60)	0.115
	III	20	0.72^∗^ (0.69–1.15)	14	0.69 (0.15–1.26)	8	0.21 (0.17–0.26)	0.189
	***P*- value**		***0.006***		0.611		0.281	
Tumor size	T2	15	1.08^∗^ (0.81–1.35)	12	0.43**^a^** (0.11–0.63)	9	0.22**^a^** (0.17–0.45)	***0.020***
	T3	65	0.58 (0.44–0.73)	30	0.53 (0.30–1.26)	18	0.21**^ab^** (0.20–0.30)	***0.011***
	T4	19	0.63 (0.47–0.79)	6	0.55 (0.14–1.26)	5	0.68 (0.28–1.08)	0.368
	***P*- value**		***0.008***		0.291		0.560	
Lymph node	Negative	41	0.71^∗^ (0.53–0.89)	26	0.55 (0.16–0.92)	22	0.30 (0.17–0.60)	0.236
	Positive	39	0.54 (0.41–0.68)	22	0.43 (0.17–0.69)	10	0.25 (0.20–0.43)	0.050
	***P*- value**		***0.006***		0.885	0.920		
Metastasis	Negative	71	0.67^∗^ (0.50–0.84)	38	0.55 (0.15–1.26)	24	0.28 (0.20–0.60)	0.050
	Positive	31	0.51 (0.38–0.64)	10	0.38 (0.30–0.63)	8	0.24 (0.16–0.30)	0.178
	***P*- value**		***0.005***		0.696		0.364	
	II	32	0.71^∗^ (0.53–0.89)	20	0.51 (0.15–0.92)	13	0.20 (0.14–0.60)	0.472
Stage	III	39	0.59 (0.44–0.74)	18	0.58 (0.28–1.26)	11	0.36 (0.21–1.08)	0.097
	IV	31	0.54 (0.41–0.68)	10	0.38 (0.30–0.63)	8	0.24 (0.16–0.30)	0.178
	**P- value**		***0.019***		0.825		0.346	

*Data presented as medians and interquartile range (IQR) of 5mC level (ng/DNA) of CRC patients at baseline and after treatment with FP therapy for 3 and 6 months. Significant *P*- values were with bold italic font. **^∗^**superscript is a significant difference (*P*-value < 0.05) when the two CRC subgroups were compared by Mann–Whitney. **^*a*^**Is a significant difference (*P*-value < 0.05) when the 3 and 6 months treated CRC patients were compared with their baseline by Wilcoxon Matched. **^*b*^**Is a significant difference (*P*-value < 0.05) when the 6 months treated CRC patients were compared with their 3 months level by Wilcoxon Matched. 5mC, 5 methoxy 2 deoxy cytosine; CEA, carcinoembyonic antigen; CA19.9, carbohydrate antigen 19.9; NA, not applicable.*

Insignificant differences in 5mC % among the different subgroups of CRC patients prior FP therapy, whereas FP therapy for 6 months caused significant reduction in 5mC % in patients with T2 tumors and negative lymph nodes compared to the baseline and the 3 months level, **Table 2**.

**Table 2:** Global 5mC% in subgroups of patients at baseline and after treatment with FP based therapy.

Variables		5mC % in CRC patients						*P*-value
		At baseline		After 3 months of FP therapy		After 6 months of FP therapy		
			
		*N*	Median (IQR)	*N*	Median (IQR)	*N*	Median (IQR)	
Age	≤45	47	5.15 (2.44–13.49)	23	6.35 (5.92–14.78)	15	5.45 (5.34–5.87)	0.223
	>45	55	8.62 (5.52–4.08)	25	22.67 (9.80–35.70)	17	5.48 (5.02–6.35)	0.097
	***P*-value**	0.117		0.126		0.602		
Sex	Female	47	5.72 (2.44–13.49)	20	6.53 (6.22–14.78)	13	5.68 (3.88–6.11)	0.097
	Male	55	8.81 (5.00–21.21)	28	22.67 (9.80–35.70)	19	5.40 (5.02–6.14)	0.223
	***P*-value**	0.403		0.409		0.831		
CEA level	Normal	46	15.44 (11.58–19.30)	5	6.55 (6.15–35.7)	4	5.87 (2.27–6.35)	0.097
	High	33	9.58 (7.19–11.98)	13	33.65(10.96–67.23)	8	5.02 (3.11–5.48)	0.368
	***P*-value**	0.985		0.353		0.408		
CA19.9 level	Normal	57	15.21 (11.41–19.01)	2	12.87 (6.5–33.65)	1	5.48 (5.48–5.48)	NA
	High	15	9.80 (7.35–12.25)	17	51.47(35.76–67.23)	11	5.02 (4.22–11.02)	0.062
	***P*-value**	0.818		0.080		0.472		
Tumor location	Right colon	30	13.52 (10.14–16.90)	15	22.67 (14.78–33.65)	16	5.34 (5.13–7.45)	0.135
	Left colon	25	9.35 (7.01–11.69)	15	20.96 (6.19–51.47)	6	5.11 (4.32–6.22)	0.760
	Rectum	40	9.46 (7.10–11.83)	18	8.18 (6.5–10.96)	10	6.25 (6.02–7.88)	0.060
	***P*-value**	0.180		0.350		0.370		
Pathology	Adenocarcinoma	66	10.36 (7.77–12.95)	32	14.78 (9.80–35.70)	22	5.40 (4.07–5.68)	0.05
	Mucinous	34	35.15 (10.36–40.94)	16	6.36 (6.15–22.67)	10	6.25 (6.14–6.35)	0.368
	***P*-value**	0.350		0.239		0.117		
Tumor grade	II	82	10.57 (8.43–17.71)	34	10.96 (6.22–35.70)	24	5.48 (5.34–6.14)	0.097
	III	20	12.05 (9.04–15.06)	14	10.64(6.21–35.35)	8	5.02 (2.27–6.35)	0.223
	***P*-value**	0.796		0.794		0.425		
Tumor size	T2	15	9.52 (7.14–16.90)	12	22.67 (14.78–67.23)	9	3.86**^ab^** (2.27–5.45)	***0.029***
	T3	65	13.01 (6.76–22.26)	30	10.38 (6.22–35.70)	18	5.48 (5.02–6.35)	0.097
	T4	19	8.14 (7.61–19.68)	6	6.35(6.15–6.55)	5	5.87 (3.87–6.87)	0.368
	***P*-value**	0.819		0.230		0.407		
Lymph node	Negative	41	13.52 (10.14–16.90)	26	14.78 (6.55–49.73)	22	5.45**^ab^** (5.02–5.87)	***0.022***
	Positive	39	9.46(7.10–11.83)	22	8.56 (5.92–22.67)	10	6.14 (4.14–7.14)	0.789
	***P*-value**	0.350		0.157		0.384		
Metastasis	Negative	71	8.52 (4.14–16.90)	38	14.78 (6.22–35.70)	24	5.45 (3.11–6.14)	0.050
	Positive	31	6.69 (5.27–12.11)	10	8.73 (3.95–39.10)	8	5.48 (5.02–6.35)	0.368
	***P*-value**	0.909		0.695		0.732		
Stage	II	32	13.52 (10.14–16.90)	20	24.22 (9.80–49.73)	13	5.34 (3.11–5.45)	0.097
	III	39	9.80 (7.35–12.25)	18	6.55 (6.15–22.67)	11	6.01 (5.87–6.14)	0.368
	IV	31	9.80 (7.35–12.25)	10	8.73 (3.95–39.10)	8	5.48 (5.02–6.35)	0.368
	***P*-value**	0.706		0.488		0.446		

*Data presented as medians and interquartile range (IQR) of 5mC% calculated as [5mC (ng)/(2dC (ng) + 5mC (ng)] ^∗^100. Significant *P*-values were with bold italic font. **^∗^**Superscript is a significant difference (*P*-value < 0.05) when two CRC subgroups were compared by Mann–Whitney. **^*a*^**Is a significant difference (*P*-value < 0.05) when the 3 and 6 months treated CRC patients were compared with their baseline by Wilcoxon Matched. **^*b*^**Is a significant difference (*P*-value < 0.05) when the 6 months treated CRC patients were compared with their 3 months level by Wilcoxon Matched. 5mC, 5 methoxy 2 deoxy cytosine; CEA, carcinoembyonic antigen; CA19.9, carbohydrate antigen 19.9; NA, not applicable.*

### DNMT3A and DNMT3B Genes Expression in Subgroups of CRC Patients

Subgroup analysis of CRC patient after 3 months of FP therapy showed that DNMT3A expression level was significantly high in patients with adenocarcinoma type of tumors and high CA19.9 level. Pairwise comparison, FP therapy revealed that significant induction in DNMT3A gene expression was associated CRC patients with good prognostic features after 3 months of therapy. CRC patients with right colon tumors, adenocarcinoma, negative lymph nodes, negative metastasis and stage II had significant higher 5mC% compared to their baseline after 3 months of FP therapy, **Table 3**.

**Table 3:** DNMT3A gene expression in subgroups of patients at baseline and after treatment with FP based therapy.

Variables		DNMT3A gene expression relative to B- actin (2^-ΔCT)^ in CRC patients						*P*-value
		At baseline		After 3 months		After 6 months		
				of FP therapy		of FP therapy		
			
		*N*	Median × 10^3^ (IQR)	*N*	Median × 10^3^ (IQR)	*N*	Median × 10^3^ (IQR)	
Age	≤45	47	1.28 (0.033–3.83)	23	1.26 (0.25–5.33)	15	1.224 (0.009–2.60)	0.150
	>45	55	1.07 (0.10–2.26)	25	2.62 (0.73–4.77)	17	2.003 (0.44–3.76)	0.157
	***P*-value**	0.413		0.964		0.101		
Sex	Female	47	0.81 (0.07–2.46)	20	1.26 (0.66–4.58)	13	1.19 (0.15–2.09)	0.246
	Male	55	1.20 (0.21–5.07)	28	2.64 (0.25–5.10)	19	2.59 (1.224–7.64)	0.779
	***P*-value**	0.269		0.816		0.150		
CEA level	Normal	46	1.03 (0.09–2.469)	5	2.42 (1.20–0.67)	4	1.42 (0.15–3.36)	0.486
	High	33	0.55 (0.08–4.59)	13	1.26 (0.19–5.33)	8	1.58 (1.43–7.60)	0.247
	***P*-value**	0.905		0.881		0.670		
CA19.9 level	Normal	57	1.03 (0.09–2.94)	2	1.26 (0.35–4.35)	1	1.43 (0.13–4.32)	NA
	High	15	0.18 (0.03–6.37)	17	7.79**^∗^** (6.79–63.52)	11	3.27 (2.26–6.07)	0.223
	***P*-value**	0.942		***0.025***		0.172		
Tumor location	Right colon	30	1.15 (0.24–2.94)	15	3.22**^a^** (2.42–5.72)	16	2.60 (0.81–3.35)	***0.046***
	Left colon	25	1.94 (0.29–4.12)	15	1.94 (1.01–3.53)	6	1.22 (0.44–2.26)	0.819
	Rectum	40	0.58 (0.04–2.53)	18	0.49 (0.02–4.50)	10	1.35 (0.11–3.76)	0.368
	***P*-value**	0.180		0.089		0.439		
Pathology	Adenocarcinoma	66	1.03 (0.04–2.01)	32	3.10**^∗a^** (2.95–5.72)	22	1.66 (1.01–2.76)	***0.005***
	Mucinous	34	1.72 (0.26–3.01)	16	0.96 (0.07–2.62)	10	1.12 (0.06–4.93)	0.301
	***P*-value**	0.263		***0.020***		0.340		
Tumor grade	II	82	1.03 (0.07–2.95)	34	2.64 (0.42–4.88)	24	1.53 (0.19–3.76)	0.200
	III	20	2.06 (0.09–6.15)	14	1.84 (0.34–4.24)	8	1.22 (0.073–3.27)	0.156
	***P*-value**	0.386		0.910		0.628		
Tumor size	T2	15	1.94 (0.041–5.47)	12	2.71 (1.81–9.35)	9	2.59 (0.04–2.61)	0.247
	T3	65	0.98 (0.04–2.86)	30	2.58 (0.42–4.88)	18	1.59 (1.01–7.64)	0.097
	T4	19	1.51 (0.18–2.77)	6	0.62 (0.003–2.87)	5	0.05 (0.001–1.26)	0.472
	***P*-value**	0.337		0.081		0.150		
Lymph node	Negative	41	1.01 (0.06–2.77)	26	2.92**^a^** (2.67–4.67)	22	1.19 (0.116–2.17)	***0.007***
	Positive	39	1.51 (0.11–3.61)	22	2.42 (0.03–5.11)	10	3.27 (1.43–7.60)	0.988
	***P*-value**	0.343		0.628		0.055		
Metastasis	Negative	71	1.03 (0.04–3.01)	38	2.62**^a^** (0.42–5.33)	24	1.61 (0.73–3.27)	***0.048***
	Positive	31	1.12 (0.10–4.06)	10	1.25 (0.42–4.87)	8	1.43 (0.07–3.76)	0.846
	***P*-value**	0.710		0.608		0.671		
	II	32	1.09 (0.03–2.74)	20	3.62**^a^** (2.96–5.22)	13	1.06 (0.15–2.17)	***0.028***
Stage	III	39	0.91 (0.06–3.51)	18	2.62 (0.07–5.39)	11	2.93 (1.35–3.51)	0.717
	IV	31	1.12 (0.14–4.06)	10	1.25 (0.42–4.87)	8	1.43 (0.07–3.76)	0.846
	***P*-value**	0.755		0.875		0.115		

*Data presented as medians ×**10^*3*^** and interquartile range (IQR) of DNMT3A expression level relative to B- actin (2^-ΔCT^). Significant *P*-values were with bold italic font. **^∗^**Superscript is a significant difference (*P*-value < 0.05) when two CRC subgroups was compared by Mann–Whitney. **^*a*^**Is a significant difference (*P*-value < 0.05) when the 3 and 6 months treated patients were compared with their baseline by Wilcoxon Matched. DNMT3A, DNA- methyltransferase 3A; CEA, carcinoembyonic antigen; CA19.9, carbohydrate antigen 19.9; NA, not applicable.*

A significant DNMT3B overexpression was seen in patients with mucinous and grade III tumors baseline. FP therapy increased significantly DNMT3B expression in patients with high CA19.9 after 3 months and in patients with negative metastasis and stage II after 6 months of therapy, **Table 4**.

**Table 4:** DNMT3B gene expression in subgroups of patients at baseline and after treatment with FP therapy.

Variables		DNMT3B gene expression relative to B- actin (2^-ΔCT)^ in CRC patients						*P*-value
		At baseline		After 3 months		After 6 months		
				of FP therapy		of FP therapy		
			
		*N*	Median(IQR)	*N*	Median(IQR)	*N*	Median(IQR)	
Age	≤45	47	0.80 (0.45–11.31)	23	0.57 (0.11–2.04)	15	0.66 (0.48–2.43)	0.236
	>45	55	0.60 (0.15–4.23)	25	0.60 (0.30–2.20)	17	0.66 (0.11–0.90)	0.529
	***P*-value**		0.133		0.628		0.245	
Sex	Female	47	0.70 (0.41–10.93)	20	0.30 (0.18–0.86)	13	0.70 (0.59–1.59)	0.236
	Male	55	0.64 (0.25–4.23)	28	0.80 (0.37–2.51)	19	0.55 (0.12–1.32)	0.178
	***P*-value**		0.271		0.092		0.355	
**CEA Level**	Normal	46	0.62 (0.27–9.92)	5	0.70 (0.21–2.14)	4	0.59 (0.18–0.81)	0.307
	High	33	0.50 (0.15–11.24)	13	0.83 (0.10–2.51)	8	1.71 (0.11–1.73)	0.998
	***P*-value**		0.935		0.503		0.786	
CA19.9 level	Normal	57	0.70 (0.21–11.31)	2	0.83 (0.48–2.20)	1	0.68 (0.68–0.68)	NA
	High	15	0.80 (0.40–20.82)	17	2.51**^∗^** (2.51–4.71)	11	0.62 (0.20–1.59)	0.980
	***P*-value**		0.699		***0.010***		0.794	
Tumor location	Right colon	30	0.80 (0.30–4.23)	15	0.37 (0.11–0.70)	16	0.64 (0.20–0.90)	0.459
	Left colon	25	0.30 (0.15–0.87)	15	1.42 (0.20–3.30)	6	1.59 (0.11–24.93)	0.264
	Rectum	40	1.29 (0.41–29.05)	18	0.72 (0.36–2.21)	10	0.70 (0.13–1.71)	0.066
	***P*-value**		0.08		0.379		0.615	
Pathology	Adenocarcinoma	66	0.60 (0.25–3.10)	32	0.70 (0.15–2.09)	22	0.68 (0.20–0.90)	0.584
	Mucinous	34	0.99**^∗^** (0.40–11.24)	16	0.47 (0.26–1.58)	10	0.48 (0.13–5.17)	0.276
	***P*-value**		***0.020***		0.899		0.881	
Tumor grade	II	82	0.66 (0.27–4.44)	34	0.75 (0.30–2.14)	24	0.66 (0.16–1.25)	0.247
	III	20	1.39**^∗^** (0.20–11.24)	14	0.39 (0.18–0.60)	8	0.66 (0.48–5.17)	0.819
	***P*-value**		***0.040***		0.500		0.342	
Tumor size	T2	15	0.56 (0.35–6.32)	12	0.49 (0.11–2.14)	9	0.59 (0.20–0.62)	0.717
	T3	65	0.80 (0.25–8.06)	30	0.57 (0.30–2.04)	18	0.81 (0.20–1.73)	0.395
	T4	19	0.49 (0.15–0.87)	6	0.70 (0.10–4.08)	5	0.18 (0.11–0.70)	0.717
	***P*-value**		0.569		0.932		0.243	
Lymph node	Negative	41	0.59 (0.20–0.62)	26	0.30 (0.11–0.80)	22	0.75 (0.40–1.66)	0.441
	Positive	39	0.81 (0.20–1.73)	22	0.86 (0.57–2.46)	10	0.48 (0.13–0.81)	0.236
	***P*-value**		0.075		0.068		0.319	
Metastasis	Negative	71	0.70 (0.29–4.77)	38	0.60 (0.30–2.04)	24	0.75 (0.48–1.71)	0.465
	Positive	31	0.50 (0.19–6.16)	10	0.37 (0.10–2.51)	8	0.11**^∗^** (0.10–0.18)	0.097
	***P*-value**		0.196		0.456		***0.014***	
	II	32	0.60 (0.20–3.10)	20	0.30 (0.18–0.80)	13	0.85 (0.59–1.73)	0.641
Stage	III	39	1.19 (0.33–9.92)	18	1.41 (0.57–2.46)	11	0.68 (0.31–1.26)	0.368
	IV	31	0.50 (0.19–6.16)	10	0.37 (0.10–2.51)	8	0.11**^∗^** (0.10–0.18)	0.097
	***P*-value**		0.271		0.196		***0.037***	

*Data presented as medians and interquartile range (IQR) of DNMT3B expression level relative to B- actin (2^-Δ*CT*^). Significant *P*-values were with bold italic font. **^∗^**Superscript is a significant difference (*P*-value < 0.05) when two CRC subgroups were compared by Mann–Whitney. DNMT3B, DNA- methyltransferase 3B; CEA, carcinoembyonic antigen; CA19.9, carbohydrate antigen 19.9; NA, not applicable.*

### Univariate Analysis of OS

Kaplan–Meier analysis revealed a significant higher OS in patients with normal initial level of CEA (30.6 versus15.13 months) and CA19.9 (28.22 versus 12.7 months), **Supplementary Figures S2A,B**, respectively. Significant improvement in OS was shown in patients with negative metastasis (30.6 versus 12.33 months), **Supplementary Figure S2C** and patients in stages II and III (27.46 and 25.23 months versus 12.33 months), **Supplementary Figure S2D**.

**FIGURE 3:**
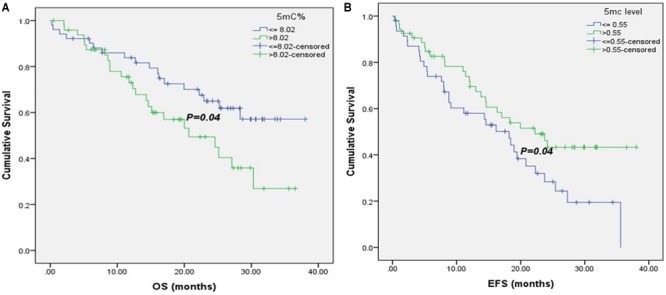
Kaplan–Meier survival curves for CRC patients according to their global DNA methylation levels. **(A)** OS of CRC patients with 5mC% ≤ 8.02 and >8.02. **(B)** EFS of CRC patients with 5mC level ≤ 0.55 and >0.55.

Moreover, it was noticed that patients with 5mC % ≤8.02 % (8.02 is the median 5mC % in 102 baseline CRC patients) had significant higher median OS (26.07 versus 23.3 months), as shown in **Figure 3A**.

### Univariate Analysis of EFS

There was a significant association of higher median EFS in patients with normal CEA and CA19.9 baseline levels (35.6 versus 14.35 months, for CEA and 23.73 versus 9.1 months, for CA19.9), **Supplementary Figures S3A,B**. Also, patients with T3 tumors had significant higher EFS (35.6 months) than those with T4 tumors (19.43 months), **Supplementary Figure S3C**. Negative metastatic patients had significant improvement in their EFS (35.6 versus 8.1 months), **Supplementary Figure S3D**, as well as patients at stages II and III (30.95 and 24.17 months, respectively versus 16.47 months for patients at stage IV), **Supplementary Figure S3E**. Moreover, it was shown that patients with 5mC level > 0.55 (0.55 is the median 5mC level in 102 baseline CRC patients) had significant higher EFS (22.23 versus18.2 months), **Figure 3B**.

### Multivariate Analysis of Patients‘ Survival

**FIGURE 4:**
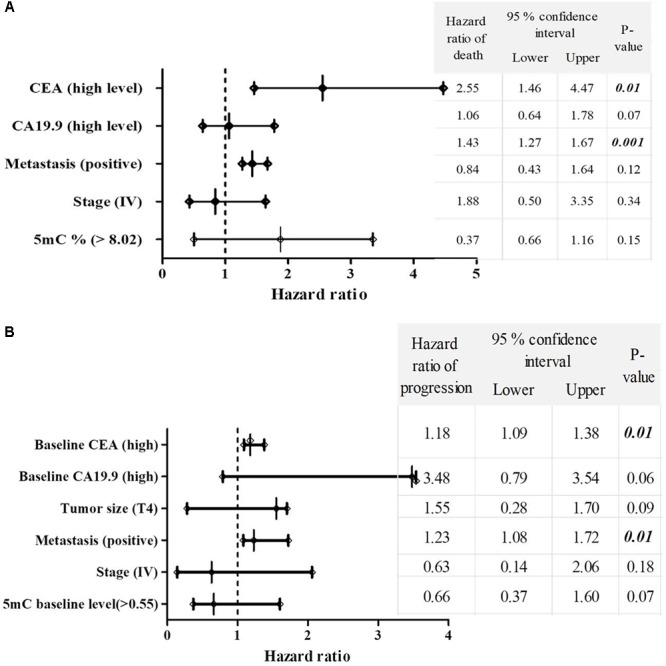
The multivariate COX regression hazard model. **(A)** Hazard ratio of death and **(B)** progression. Table indicates the hazard ratio and 95% confidence interval, associated with high level of CEA, high level of CA19.9, T4, positive metastasis, stage (IV) and 5mc level > 0.55. *P*-value < 0.05 is a significant hazard of death or progression, when hazard ratios of table variables compared with reference variables. **Reference variables (hazard value of 1):** low level of CEA, low level of CA19.9, T2 and T3 tumors, negative metastasis, stages II and III and 5mC level ≤ 0.55. OS, overall survival; EFS, event free survival; CEA, carcinoembyonic antigen; CA19.9, carbohydrate antigen 19.9; 5mC, 5, methoxy 2 deoxy cytosine.

Significant increase in the hazard of death and progression were associated CRC patients with high CEA level and positive metastasis, as shown in **Figures 4A,B**.

## Discussion

CRC persists as one of the most prevalent and deadly tumor type in both men and women worldwide, in spite of widespread effective measures of preventive screening and major advances in treatment options (Arnold et al., 2017). Epigenetics especially DNA methylation is one of the contributing factors to CRC pathogenesis and treatment outcome (Moore et al., 2013).

Methylation of the C-5 position of cytosine in genomic DNA is a central mammalian epigenetic control mechanism that affects gene expression (Moore et al., 2013). In this study, the change in global DNA methylation in terms of 5mC level and 5mC % at baseline as well as after FP therapy was investigated in the peripheral blood samples. In this work peripheral blood lymphocytes was preferred as a sampling source than the tissue sections of individual tumor because of the epigenetic intratumoral heterogeneity and differences in DNA methylation pattern (Martínez-Cardús et al., 2016; Jones et al., 2017). In addition, blood-based specimens such DNA extracted from leukocytes are considered as a non-invasive sampling (Li et al., 2012). Moreover, Barciszewska et al. (2014) found the same level of hypomethylation in DNA of tumor tissue and peripheral blood samples from patients with primary and metastatic tumors. Furthermore, it was shown that DNA methylation signatures in peripheral blood for multiple disease-related genes are strongly linked to mortality outcomes (Zhang et al., 2017).

In this study, CRC patients at baseline had significant global hypomethylation, in terms of decreased median levels of 5mC and 5mC % compared to healthy control. This hypomethylation of DNA facilitates the aberrant expression of protooncogenes/oncogenes and potentially stimulates tumor cell proliferation (Kushwaha et al., 2016). It was suggested that DNA hypomethylation is likely to induce a cascade effect with direct implications in the determination of the progression pathway, and hence the patient’s outcome (Rodriguez et al., 2006). One of the possible mechanisms for regulation of DNA methylation in cancer is the ischemic/hypoxic condition found in solid tumors. It was found that 5-mC levels altered with hypoxia. It was suggested that hypomethylation occurs via an active process or through lack of maintenance methylation (Shahrzad et al., 2007).

In agreement with our results, DNA global hypomethylation was detected in both colonic and leukocyte of patients with CRC (Li et al., 2014; Tian et al., 2017). In addition, an increased risk of a number of cancers, including colorectal adenoma was associated with lower level of global methylation in peripheral blood (Lim et al., 2008; Barciszewska et al., 2014). On contrary, Nan et al. (2013) reported no evidence of hypomethylation of leukocyte genomic DNA and increased risk of CRC among women. In addition, Jiang et al. (2017) showed insignificant difference in 5mC overall methylation between the cancerous and precancerous tissues of the colon.

Although the state of hypomethylation observed in CRC patients in this study, an overexpression of DNMT3A and DNMT3B genes were observed. It was proposed that DNA hypomethylation occurs with high expression of DNMTs because of the inhibition of *de novo* methylation through DNMT3 and post- translational degradation of the enzymes, via a class of proteins (UHRF1 and UHRF2) that have been frequently overexpressed in cancer and emerged as epigenetic regulators during DNA replication (Jia et al., 2016). It was suggested during cancer progression, an increase in DNMTs activity was seen along DNA hypomethylation, reflecting the chromosomal instability, chromosomal rearrangement of the genome (De Capoa et al., 1999). Moreover, DNMTs overexpression by 15-folds were shown in histologically normal mucosa from patients with cancers or benign polyps that can precede to cancers compared to normal colon mucosa from patients without neoplasia, 60 folds increased in the premalignant polyps, and >200 folds increased in the cancers (el-Deiry et al., 1991).

Subgroup analysis and pairwise comparison conducted for CRC patients with similar clinicopathological characters showed that within the hypomethylated CRC patients, significant higher global 5mC level was associated with T2 tumors, negative lymph nodes, and non-metastasis. In agreement, Morikawa et al. (2012) indicated that DNA methylation level is negatively associated with T-stage and is significantly reduced with lymph node metastases in CRC patients. These results were in concordance with most of research conducted on global methylation levels either by 5mC or by LINE-1 (a repetitive sequence of CpG islands across the whole genome and considered as surrogate marker for global DNA methylation) in CRC disease (Jiang et al., 2017; Tian et al., 2017). Moreover, there was statistically significant inverse relationship observed between DNA methylation in colon tissue and adenoma risk which was attributed to deregulation of genes expression causing poor tumor differentiation, altered cell cycle regulation and increasing cancer invasiveness (King et al., 2014).

Also, our patients who were >45 years showed significant higher median 5mC level. It was hypothesized that 63% clones of normal colon cells were progressively methylated in an age-dependent manner where the CpG island hypermethylation of inflammatory bowel diseases like CRC was supposed to be promoted by aging rather than inflammation (Olaru et al., 2012). Likely, significant LINE-1 hypomethylation was shown in early onset CRC than late onset (Antelo et al., 2012).

In the present study, 5mC level was significantly high in patients who had their tumors in the right side of GIT. Our result was in line with Yamauchi et al. (2012), who found that DNA methylation was gradually decreased from rectum to descending colon, and then increased from descending colon to ascending colon. In addition, it was reported that aberrant DNA methylation is more prominent in proximal compared with distal CRCs (Exner et al., 2015). However, Antelo et al. (2012) reported insignificant difference in LINE-1 global methylation between proximal and distal colonic tumors. Contrary to the above, Tian et al. (2017) observed significant high 5mC level in rectal cancer patients who had lymph node metastasis. The change in the degree of methylation along the GIT was proposed to be due to the differences in the frequencies of key molecular features such as microsatellite instability (MSI), CpG island methylator phenotype (CIMP), BRAF and PIK3CA mutations along the length of the colorectum (Yamauchi et al., 2012). In addition to microbiota that influence the immune response and inflammation from the proximal to distal colorectal segments might lead to the progression of colorectal tumors that exhibit LINE-1 hypomethylation (Mima et al., 2016).

Our study revealed significant increase in 5mC level in patients with high-grade tumors and also significant DNMT3B overexpression in patients with mucinous and high-grade tumors. In agreement with our data, Antelo et al. (2012) showed that mucinous tumors have ≥ 65 % higher LINE-1 methylation level. Interestingly, overexpression of DNMT3B in CRC mice model (Apc Min/+ mice), enhanced the number of colon tumors approximately two folds and increased the average size of colonic microadenomas, whereas DNMT3A had no effect (Linhart et al., 2007). Cancer cells have many aberrant transcripts of DNMT3B, which is linked to *E*-cadherin promoter gene methylation and CRC aggressiveness (Brambert et al., 2015). Moreover, DNMT3B overexpression was frequently associated with LINE-1 hypermethylation, high-grade histology with many features of combined high MSI, CIMP and BRAF V600E mutation and increased KRAS and wild-type p53 expressions (Hugen et al., 2015).

CRC patients treated with FP therapy, in the current study, showed significant reductions in their global DNA methylation. Global hypomethylation can be attributed to inhibition of one-carbon metabolic intermediates induced by FP therapy leading to imbalance between SAM and *S*- adenosylhomocysteine (SAH), resulting in altering normal DNA methylation patterns (Smith et al., 2008). It was suggested global DNA methylation induced by 5-FU was due to the generated reactive oxygen species, up-regulating ten-eleven translocation (TET1) expression and function. TET1 converts 5-mC to 5-hydroxymethylcytosine (5-hmC), 5-formylcytosine (5-fC), and 5-carboxylcytosine (5-caC), finally leading to cytosine (Ito et al., 2011). These modified bases may not only serve as intermediates in the DNA demethylation process but may also increase the diversity of the epigenetic states of genomic DNA and acquisition of 5-FU resistance CRC phenotype (Kang et al., 2014). Moreover the hypomethylation was due to the formation of a nucleoside analog which may inhibit DNMTs and leads to its degradation causing demethylation (Newman et al., 2015) and consequent re-expression of p16 and RASSF1A tumor suppressor genes (Castillo-Aguilera et al., 2017).

In this study, tumors with good prognostic features and higher 5mC level at baseline show better response to FP therapy. This is not only because of the high intracellular folate concentrations but also because of the silencing of critical genes like DPYD (Iacopetta et al., 2008). Moreover, variability in the DNA methylation of colonic mucosa results in different metabolic conversion of 5-FU into its active metabolite fluorouridine monophosphate (FdUMP) and inhibition of TS activity through the misincorporation of FUTP and FdUTP into RNA and DNA, respectively (Soong and Diasio, 2005).

5FU induced significant DNA hypomethylation, with significant over expression of DNMT3B after 3 months compared to their levels at baseline. The increase in DNMT3B expression was suggested to be as a compensatory mechanism for the global hypomethylation levels. DNMT3B causes hypermethylation of promoter CpG-rich regions in a variety of malignancies (Yun et al., 2012) and repress transcription in either methylation-dependent manner (Linhart et al., 2007; Nosho et al., 2009) or through their histone deacetylase activity (Fuks et al., 2001). Moreover, DNMT3B overexpression was frequently associated with LINE-1 hypermethylation, high-grade histology with many features of combined high MSI, CIMP and BRAF V600E mutation and increased KRAS and wild-type p53 expressions (Hugen et al., 2015).

In Kugimiya et al. (2015), it was seen that 5FU enhanced c-MYC activity and induced ATP-binding cassette transporters expression. DNMT3B expression is dependent on oncogenic MYC levels, which is as a site-specific transcription factor regulator for hundreds of genes (Poole et al., 2017). Increased DNMT activity is significantly associated with high rates of methylation-dependent gene silencing which contributes to 5FU resistance (Sandhu et al., 2012).

Also, we found that CRC patients with high CA19.9 exhibited significant DNMT3A and DNMT3B overexpression. However, this result is not reliable and could be attributed to chance as the number of CRC patients with normal CA19.9 was two compared to 17 patients with high CA19.9, so that association should be repeated in a larger number of patients.

The bad prognostic behavior of high CA19.9 was seen in our patients’ OS and EFS. In this study, CRC patients with high initial CEA and CA19.9 levels faced significant reduction in their OS and EFS times and also had significant increase in the hazards of both death and disease progression. Similarly, it was found that high preoperative CEA level had a negative survival impact regardless of tumor stage and the combination measurement of both tumor markers was suggested to improve the prognostic power of CRC patients‘ survival (Chen et al., 2005).

Adding to the above, patients with tumor sizes T2 and T3 had significant high EFS as well as the early stage of the disease and non- metastatic CRC patients had significant improved OS and EFS. Metastasis caused significant increase in the risk of death (hazard ratio = 1.41, *P* < 0.001) and disease progression (hazard ratio = 1.43, *P* < 0.001). In addition, the pathological stage is the most important prognostic factor following surgical resection of colorectal tumors. The prognosis for early stages (I and II) is favorable overall, in contrast to the prognosis for advanced stages (III and IV) (Zeeneldin et al., 2012).

Our results showed that patients with higher global 5mC level (>0.55) had improved EFS, while patients with 5mC % (>8.02) were associated with reduced OS. Similarly, Barciszewska et al. (2007) found lower 5mC level is correlated with advanced malignancy grade in breast and colon cancer. Improved patients‘ survival with global hypermethylation levels baseline (Kaneko et al., 2016). In addition, LINE-1 hypermethylation is negatively associated with the T- stage and lymph node metastases in CRC patients (Morikawa et al., 2012). Contrary to the previous studies, in metanalysis study of Li et al. (2014) showed that genome-wide hypomethylation have significant desirable effects on the OS of patients with various types of cancers.

Tian et al. (2017) identified the 5mC ratio between cancers to normal tissues as an independent prognostic factor for patient’s outcome, the lower the ratio is, the worse survival becomes. It was proposed that the significant deterioration of EFS with 5mC level ≤ 0.55 has been correlated with genomic instability resulting in the re-expression of proto-oncogenes or imprinted genes, as well as the activation of viral and parasitic transposons. Besides, DNA hypomethylation has been demonstrated to increase the immunogenicity and immune recognition of cancer cells through the up-regulation of different molecules involved in antigen proceeding and presentation (Rodriguez et al., 2006).

The normalization of 5mC level to the total pool of cytosines as 5mC % is proposed to be a reflection to the amount and density of methylated CpG It was suggested that there is a coordinated regulation of methylation changes between the 2 types of methylation. Kushwaha et al. (2016) observed an antagonistic functions of hypo and hyper methylation in the differentiation, cell-cycle regulation and proliferation. Hypomethylation and hypermethylation usually co-exist in the same tumor but in different sequence, it had been proposed that there is cross talk between demethylation and de novo methylation pathways during tumorigenesis, making one dependent on the other (Ehrlich, 2009). For example, DNA demethylation might be used as a type of epigenetic repair to compensate for physiologically inappropriate methylation of CpG islands overlapping promoters of tumor suppressor genes. It was proposed that DNA hypomethylation might occur early in oncogenesis followed by hypermethylation as a kind of increased, compensatory de novo methylation (Pogribny et al., 1997). However, Ehrlich (2009) linked hyper- and hypo-methylation of DNA to CRC carcinogenesis in an inter-relationship rather than dependence on each other. Both hypermethylation and hypomethylation of DNA have been observed in most tested cancers but in different sequences. Many specific gene regions become hypermethylated, and some other gene regions and many non-coding DNA repeats become hypomethylated during carcinogenesis (Nishiyama et al., 2005).

## Conclusion

Egyptian CRC patients had global DNA hypomethylation and DNMT3A and DNMT3B overexpression. FP therapy caused significant hypomethylation particularly in the subgroups of CRC patients with high baseline DNA methylation and good prognostic features. Patients with 5mC % >8.02% had significant deterioration in OS and significant positive impact in EFS was associated patients with 5mC level > 0.55. Hazards of death and progression were significantly associated with high initial CEA level and positive metastasis.

## Author Contributions

MF carried the experimental work, drafting and writing the manuscript. SES and MH extracted data of the patients, and shared in writing the manuscript. SAS and AZ shared in data analyses, writing and reviewing the manuscript. HH contributed in collecting patients’ consent and sampling procedures. EE performed the statistical analyses. All the authors have read and approved the manuscript.

## Conflict of Interest Statement

The authors declare that the research was conducted in the absence of any commercial or financial relationships that could be construed as a potential conflict of interest.

## Abbreviations

CRC: colorectal cancer
DNMT: DNA methyl transferase
DPYD: dihydropyrimidine dehydrogenase
EFS: event free survival
FdUMP: fluorouridine
FdUTP: fluorodeoxyuridine triphosphate
FP: fluoropyrimidine
FUTP: fluorouridine triphosphate
OS: overall survival
TYMS: thymidylate synthase

## References

[B1] Antelo M. Balaguer F. Shia J. Shen Y. Hur K. Moreira L. (2012). A high degree of LINE-1 hypomethylation is a unique feature of early-onset colorectal cancer. *PLoS One* 9:e45357. 10.1371/journal.pone.0045357 23049789 PMC3458035

[B2] Arnold M. Sierra M. S. Laversanne M. Soerjomataram I. Jemal A. Bray F. (2017). Global patterns and trends in colorectal cancer incidence and mortality. *Gut* 66 683–691. 10.1136/gutjnl-2015-310912 26818619

[B3] Baharudin R. Ab Mutalib N.-S. Othman S. N. Sagap I. Rose I. M. Mokhtar M. (2017). Identification of predictive DNA methylation biomarkers for chemotherapy response in colorectal cancer. *Front. Pharmacol.* 8:47 10.3389/fphar.2017.00047 PMC5303736 28243201

[B4] Barciszewska A. M. Murawa D. Gawronska I. Murawa P. Nowak S. Barciszewska M. Z. (2007). Analysis of 5-methylcytosine in DNA of breast and colon cancer tissues. *IUBMB Life* 59 765–770. 18085476 10.1080/15216540701697412

[B5] Barciszewska A.-M. Nowak S. Naskrêt-Barciszewska M. Z. (2014). The degree of global DNA hypomethylation in peripheral blood correlates with that in matched tumor tissues in several neoplasia. *PLoS One* 9:e92599. 10.1371/journal.pone.0092599 24651295 PMC3961436

[B6] Brambert P. R. Kelpsch D. J. Hameed R. Desai C. V. Calafiore G. Godley L. A. (2015). DNMT3B7 expression promotes tumor progression to a more aggressive phenotype in breast cancer cells. *PLoS One* 10:e0117310. 10.1371/journal.pone.0117310 25607950 PMC4301645

[B7] Castillo-Aguilera A. O. Depreux P. Halby L. Arimondo P. Goossens L. (2017). DNA methylation targeting: the DNMT/HMT crosstalk challenge. *Biomolecules* 7:3. 10.3390/biom7010003 28067760 PMC5372715

[B8] Chen C. C. Yang S. H. Lin J. K. Lin T. C. Chen W. S. Jiang J. K. (2005). Is it reasonable to add preoperative serum level of CEA and CA19-9 to staging for colorectal cancer? *J. Surg. Res.* 124 169–174. 15820244 10.1016/j.jss.2004.08.013

[B9] Crain P. F. (1990). Preparation and enzymatic hydrolysis of DNA and RNA for mass spectrometry. *Methods Enzymol.* 193 782–790. 1706062 10.1016/0076-6879(90)93450-y

[B10] De Capoa A. Febbo F. R. Giovanelli F. Niveleau A. Zardo G. Marenzi S. (1999). Reduced levels of poly (ADP-ribosyl) ation result in chromatin compaction and hypermethylation as shown by cell-by-cell computer assisted quantitative analysis. *FASEB J.* 13 89–93. 9872933 10.1096/fasebj.13.1.89

[B11] Ehrlich M. (2009). DNA hypomethylation in cancer cells. *Epigenomics* 1 239–259. 10.2217/epi.09.33 20495664 PMC2873040

[B12] el-Deiry W. S. Nelkin B. D. Celano P. Yen R. W. Falco J. P. Hamilton S. R. (1991). High expression of the DNA methyl transferase gene characterizes human neoplastic cells and progression stages of colon cancer. *Proc. Natl. Acad. Sci. U.S.A.* 88 3470–3474. 2014266 10.1073/pnas.88.8.3470 PMC51469

[B13] Exner R. Pulverer W. Diem M. Spaller L. Woltering L. Schreiber M. (2015). Potential of DNA methylation in rectal cancer as diagnostic and prognostic biomarkers. *Br. J. Cancer* 113 1035–1045. 10.1038/bjc.2015.303 26335606 PMC4651135

[B14] Farias N. Ho N. Butler S. Delaney L. Morrison J. Shahrzad S. (2015). The effects of folic acid on global DNA methylation and colonosphere formation in colon cancer cell lines. *J. Nutr. Biochem.* 26 818–826. 10.1016/j.jnutbio.2015.02.002 25804133

[B15] Fuks F. Burgers W. A. Godin N. Kasai M. Kouzarides T. (2001). Dnmt3a binds deacetylases and is recruited by a sequence-specific repressor to silence transcription. *EMBO J.* 2001 2536–2544. 11350943 10.1093/emboj/20.10.2536 PMC125250

[B16] Hugen N. Simons M. Haliloviæ A. van der Post R. S. Bogers A. J. Marijnissen-van Zanten M. A. (2015). The molecular background of mucinous carcinoma beyond MUC2. *Clin. J. Pathol.* 1 3–17. 10.1002/cjp2.1 27499889 PMC4858120

[B17] Iacopetta B. Kawakami K. Watanabe T. (2008). Predicting clinical outcome of 5-fluorouracil-based chemotherapy for colon cancer patients: is the CpG island methylator phenotype the 5- fluorouracil responsive subgroup? *Int. JCO* 13 498–503. 10.1007/s10147-008-0854-3 19093176

[B18] Ito S. Shen L. Dai Q. Wu S. C. Collins L. B. Swenberg J. A. (2011). Tet proteins can convert 5-methylcytosine to 5-formylcytosine and 5-carboxylcytosine. *Science* 333 1300–1303. 10.1126/science.1210597 21778364 PMC3495246

[B19] Jia Y. Li P. Fang L. Zhu H. Xu L. Cheng H. (2016). Negative regulation of DNMT3A de novo DNA methylation by frequently overexpressed UHRF family proteins as a mechanism for widespread DNA hypomethylation in cancer. *Cell Discov.* 2:16007. 10.1038/celldisc.2016.7 27462454 PMC4849474

[B20] Jiang C. Buckingham L. Barbanera W. Korang Y. Bishesari F. Melson J. (2017). LINE-1 is preferentially hypomethylated within adenomatous polyps in the presence of synchronous colorectal cancer. *Clin. Epigenetics* 9 9–25. 28293326 10.1186/s13148-017-0325-7 PMC5345219

[B21] Jones H. G. Jenkins G. Williams N. Griffiths P. Chambers P. Beynon J. (2017). Genetic and epigenetic intra-tumour heterogeneity in colorectal cancer. *World J. Surg.* 41 1375–1383. 10.1007/s00268-016-3860-z 28097409 PMC5394146

[B22] Kaneko M. Kotake M. Bando H. Yamada T. Takemura H. Minamoto T. (2016). Prognostic and predictive significance of long interspersed nucleotide element-1 methylation in advanced-stage colorectal cancer. *BMC Cancer* 16:945. 10.1186/s12885-016-2984-8 27955637 PMC5154037

[B23] Kang K. Piao M. Kim K. Kang H. Chang W. Park I. (2014). Epigenetic modification of Nrf2 in 5-fluorouracil-resistant colon cancer cells: involvement of TET- dependent DNA demethylation. *Cell Death Dis.* 5:e1183. 10.1038/cddis.2014.149 24743738 PMC4001304

[B24] King W. Ashbury J. Taylor S. Tse M. Pang S. Louw J. (2014). A cross-sectional study of global DNA methylation and risk of colorectal adenoma. *BMC Cancer* 14:488. 10.1186/1471-2407-14-488 24998982 PMC4227295

[B25] Kuchiba A. Iwasaki M. Ono H. Kasuga Y. Yokoyama S. Onuma H. (2014). Global methylation levels in peripheral blood leukocyte DNA by LUMA and breast cancer: a case–control study in Japanese women. *BJC* 110 2765–2771. 10.1038/bjc.2014.223 24786600 PMC4037832

[B26] Kugimiya N. Nishimoto A. Hosoyama T. Ueno K. Enoki T. Li T.-S. (2015). The c-MYC-ABCB5 axis plays a pivotal role in 5-fluorouracil resistance in human colon cancer cells. *J. Cell. Mol. Med.* 19 1569–1581. 10.1111/jcmm.12531 25689483 PMC4511355

[B27] Kushwaha G. Dozmorov M. Wren J. D. Qiu J. Shi H. Xu D. (2016). Hypomethylation coordinates antagonistically with hypermethylation in cancer development: a case study of leukemia. *Hum. Genomics* 10:18. 10.1186/s40246-016-0071-5 27461342 PMC4965721

[B28] Leonhardt H. Page A. W. Weier H. U. Bestor T. H. (1992). A targeting sequence directs DNA methyltransferase to sites of DNA replication in mammalian nuclei. *Cell* 71 865–873. 1423634 10.1016/0092-8674(92)90561-p

[B29] Li J. Huang Q. Zeng F. Li W. He Z. Chen W. (2014). The prognostic value of global DNA hypomethylation in cancer: a meta-analysis. *PLoS One* 9:e106290. 10.1371/journal.pone.0106290 25184628 PMC4153632

[B30] Li L. Choi J.-Y. Lee K.-M. Sung H. Park S. K. Oze I. (2012). DNA methylation in peripheral blood: a potential biomarker for cancer molecular epidemiology. *J. Epidemiol.* 22 384–394. 22863985 10.2188/jea.JE20120003 PMC3798632

[B31] Lim U. Flood A. Choi S. W. Albanes D. Cross A. J. Schatzkin A. (2008). Genomic methylation of leukocyte DNA in relation to colorectal adenoma among asymptomatic women. *Gastroenterology* 134 47–55. 10.1053/j.gastro.2007.10.013 18166347 PMC2211566

[B32] Linhart H. G. Lin H. Yamada Y. Moran E. Steine E. J. Gokhale S. (2007). Dnmt3b promotes tumorigenesis in vivo by gene-specific de novo methylation and transcriptional silencing. *Genes Dev.* 21 3110–3122. 18056424 10.1101/gad.1594007 PMC2081977

[B33] Ma H. Zhang W. Hu J. Yu Z. Chen Y. Luo Q. (2009). Analysis of global DNA methylation levels in human blood using high-performance liquid chromatography/tandem electrospray ionization mass spectrometry. *Eur. J. Mass Spectrom.* 15 555–561. 10.1255/ejms.1007 19661563

[B34] Martínez-Cardús A. Moran S. Musulen E. Moutinho C. Manzano J. Martinez-Balibrea E. (2016). Epigenetic homogeneity within colorectal tumors predicts shorter relapse-free and overall survival times for patients with locoregional cancer. *Gastroenterology* 151 961–972. 10.1053/j.gastro.2016.08.001 27521480

[B35] Mima K. Nowak J. A. Qian Z. R. Cao Y. Song M. Masugi Y. (2016). Tumor LINE-1 methylation level and colorectal cancer location in relation to patient survival. *Oncotarget* 7 55098–55109. 10.18632/oncotarget.10398 27391152 PMC5342404

[B36] Moore L. D. Le T. Fan G. (2013). DNA methylation and its basic function. *Neuropsychopharmacology* 38 23–38. 10.1038/npp.2012.112 22781841 PMC3521964

[B37] Morikawa T. Kuchiba A. Qian Z. R. Mino-Kenudson M. Hornick J. L. Yamauchi M. (2012). Prognostic significance and molecular associations of tumor growth pattern in colorectal cancer. *Ann. Surg. Oncol.* 19 1944–1953. 10.1245/s10434-011-2174-5 22189472 PMC3321113

[B38] Nan H. Giovannucci E. L. Wu K. Selhub J. Paul L. Rosner B. (2013). Pre-diagnostic leukocyte genomic DNA methylation and the risk of colorectal cancer in women. *PLoS One* 8:e59455. 10.1371/journal.pone.0059455 23560049 PMC3613344

[B39] Newman E. M. Morgan R. J. Kummar S. Beumer J. H. Blanchard M. S. Ruel C. (2015). A phase I, pharmacokinetic, and pharmacodynamic evaluation of the DNA methyltransferase inhibitor 5-fluoro-20-deoxycytidine, administered with tetrahydrouridine. *Cancer Chemother. Pharmacol.* 75 537–546. 10.1007/s00280-014-2674-7 25567350 PMC4344391

[B40] Nishiyama R. Qi L. Lacey M. Ehrlich M. (2005). Both hypomethylation and hypermethylation in a 0.2-kb region of a DNA repeat in cancer. *Mol. Cancer Res.* 3 617–626. 16317087 10.1158/1541-7786.MCR-05-0146 PMC1420408

[B41] Nosho K. Shima K. Irahara N. Kure S. Baba Y. Kirkner G. J. (2009). DNMT3B expression might contribute to CpG island methylator phenotype in colorectal cancer. *Clin. Cancer Res.* 15 3663–3671. 10.1158/1078-0432.CCR-08-2383 19470733 PMC2866637

[B42] Ogino S. Nosho K. Kirkner G. J. Kawasaki T. Chan A. T. Schernhammer E. S. (2008). A cohort study of tumoral LINE-1 hypomethylation and prognosis in colon cancer. *J. Natl. Cancer Inst.* 100 1734–1738. 10.1093/jnci/djn359 19033568 PMC2639290

[B43] Okano M. Xie S. Li E. (1998). Cloning and characterization of a family of novel mammalian DNA (cytosine-5) methyltransferases. *Nat. Genet.* 19 219–220. 9662389 10.1038/890

[B44] Olaru A. Cheng Y. Agarwal R. Yang J. David S. Abraham J. (2012). Unique patterns of CpG island methylation in inflammatory bowel disease-associated colorectal cancers. *Inflamm. Bowel Dis.* 18 641–648. 10.1002/ibd.21826 21830278 PMC3214229

[B45] Pogribny I. P. Miller B. J. James S. J. (1997). Alterations in hepatic p53 gene methylation patterns during tumor progression with folate/methyl deficiency in the rat. *Cancer Lett.* 115 31–38. 9097976 10.1016/s0304-3835(97)04708-3

[B46] Poole C. J. Zheng W. Lodh A. Yevtodiyenko A. Liefwalker D. Li H. (2017). DNMT3B overexpression contributes to aberrant DNA methylation and MYC-driven tumor maintenance in T-ALL and Burkitt’s lymphoma. *Oncotarget* 8 76898–76920. 10.18632/oncotarget.20176 29100357 PMC5652751

[B47] Rhee I. Bachman K. E. Park B. H. Jair K. W. Yen R. W. Schuebel K. E. (2002). DNMT1 and DNMT3b cooperate to silence genes in human cancer cells. *Nature* 416 552–556. 11932749 10.1038/416552a

[B48] Rodriguez J. Frigola J. Vendrell E. Risques R.-A. Fraga M. F. Morales C. (2006). Chromosomal instability correlates with genome-wide dna demethylation in human primary colorectal cancers. *Cancer Res.* 66 8462–8468. 16951157 10.1158/0008-5472.CAN-06-0293

[B49] Sandhu R. Rivenbark A. G. Coleman W. B. (2012). Enhancement of chemotherapeutic efficacy in hypermethylator breast cancer cells through targeted and pharmacologic inhibition of DNMT3b. *Breast Cancer Res. Treat.* 131 385–399. 10.1007/s10549-011-1409-2 21359954

[B50] Schulze I. Rohde C. Scheller-Wendorff M. Krause A. Herbst F. Riemke P. (2016). Increased DNA methylation of Dnmt3b-targets impairs leukemogenesis. *Blood* 127 1575–1586. 10.1182/blood-2015-07-655928 26729896

[B51] Shahrzad S. Bertrand K. Minhas K. Coomber B. (2007). Induction of DNA hypomethylation by tumor hypoxia. *Epigenetics* 2 119–125. 17965619 10.4161/epi.2.2.4613

[B52] Sinicrope F. A. Foster N. R. Thibodeau S. N. Marsoni S. Monges G. Labianca R. (2011). DNA mismatch repair status and colon cancer recurrence and survival in clinical trials of 5-fluorouracil-based adjuvant therapy. *J. Natl. Cancer Inst.* 103 863–875. 10.1093/jnci/djr153 21597022 PMC3110173

[B53] Smith A. D. Kim Y. I. Refsum H. (2008). Is folic acid good for everyone? *Am. J. Clin. Nutr.* 87 517–533. 18326588 10.1093/ajcn/87.3.517

[B54] Soong R. Diasio R. B. (2005). Advances and challenges in fluoropyrimidine pharmacogenomics and pharmacogenetics. *Pharmacogenomics* 6 835–847. 16296946 10.2217/14622416.6.8.835

[B55] Subramaniam D. Thombre R. Dhar A. Anant S. (2014). DNA methyltransferases: a novel target for prevention and therapy. *Front. Oncol.* 4:80. 10.3389/fonc.2014.00080 24822169 PMC4013461

[B56] Tian Y.-P. Lin A.-F. Gan M.-F. Wang H. Yu D. Lai C. (2017). Global changes of 5-hydroxymethylcytosine and 5-methylcytosine from normal to tumor tissues are associated with carcinogenesis and prognosis in colorectal cancer. *J. Zhejiang Univ. Sci. B* 18 747–756.

[B57] Yamauchi M. Morikawa T. Kuchiba A. Imamura Y. Qian Z. R. Nishihara R. (2012). Assessment of colorectal cancer molecular features along bowel subsites challenges the conception of distinct dichotomy of proximal versus distal colorectum. *Gut* 61 847–854. 10.1136/gutjnl-2011-300865 22427238 PMC3345105

[B58] Yang J. Wei X. Wu Q. Xu Z. Gu D. Jin Y. (2011). Clinical significance of the expression of DNA methyltransferase proteins in gastric cancer. *Mol. Med. Rep.* 4 1139–1143. 10.3892/mmr.2011.578 21887466

[B59] Yun J. Song S. H. Park J. Kim H. P. Yoon Y. K. Lee K. H. (2012). Gene silencing of EREG mediated by DNA methylation and histone modification in human gastric cancers. *Lab. Invest.* 92 1033–1044. 10.1038/labinvest.2012.61 22508389

[B60] Zeeneldin A. A. Saber M. M. Seif El-din I. A. Frag S. A. (2012). Colorectal carcinoma in Gharbiah district, Egypt: comparison between the elderly and non-elderly. *J. Solid Tumors* 2 13–23.

[B61] Zhang Y. Wilson R. Heiss J. Breitling L. P. Saum K.-U. Schöttker B. (2017). DNA methylation signatures in peripheral blood strongly predict all-cause mortality. *Nat. Commun.* 8:14617. 10.1038/ncomms14617 28303888 PMC5357865

