# Supplementary material for: Impact of Global DNA Methylation in Treatment Outcome of Colorectal Cancer Patients
Source: Front Pharmacol. 2018 Oct 18;9:1173. doi: 10.3389/fphar.2018.01173 (PMC6201055; doi:10.3389/fphar.2018.01173)
Supplement: Supplementary file 1 [file Table_1.DOCX]

**Table S (1): Patients` clinic-pathological characteristics and treatment protocol:**

| **Variables** | **N (%)** |
| --- | --- |
| Total number of patients | 102 (100) |
| **Age** |  |
| ≤45 years  >45 years  Ratio (young: old) 1:1.2  Median age 46  Range 19-72  Mean age: 45± 13.7 | 47 (46.07)  55 (53.92) |
| **Gender** |  |
| Male | 55 (53.92) |
| Female | 47 (46.07) |
| Ratio male: female 1: 0.85 |  |
| **Performance status** |  |
| I | 85 (83.33) |
| II | 15 (14.70) |
| III | 2 (1.96) |
| **CEA level** |  |
| Normal | 46 (58.22) |
| High | 33 (41.77) |
| **CA19.9 level** |  |
| Normal | 57 (79.16) |
| High | 15 (20.83) |
| **Tumor location** |  |
| Right colon | 30 (29.41) |
| Left colon | 25 (24.50) |
| Rectum | 40 (39.21) |
| **Pathology** |  |
| Adenocarcinoma | 71 (69.60) |
| Mucinous & signet ring | 31 (30.39) |
| **Grade** |  |
| II | 82 (80.39) |
| III | 20 (19.60) |
| **Tumor size** |  |
| T2 | 15 (14.70) |
| T3 | 65 (63.72) |
| T4 | 19 (18.62) |
| **Lymph nodes** |  |
| Negative | 41 (40.19) |
| Positive | 39 (38.23) |
| **Metastasis** |  |
| No | 71 (69.90) |
| Yes | 31 (30.39) |
| **Stage** |  |
| II | 32 (31.37) |
| III | 39 (38.23) |
| IV | 31 (30.39) |

Data presented as counted number of patients and percentage N (%) to the total number of patients (102). ++ More than one event per patient was recorded. **Abbreviations:** CEA: Carcinoembyonic antigen, CA19.9: Carbohydrate antigen 19.9, FP therapy: Fluoropyrimidine therapy.

**Figure S (1):** 5mC level correlation with DNMT3A expression in baseline CRC patients.


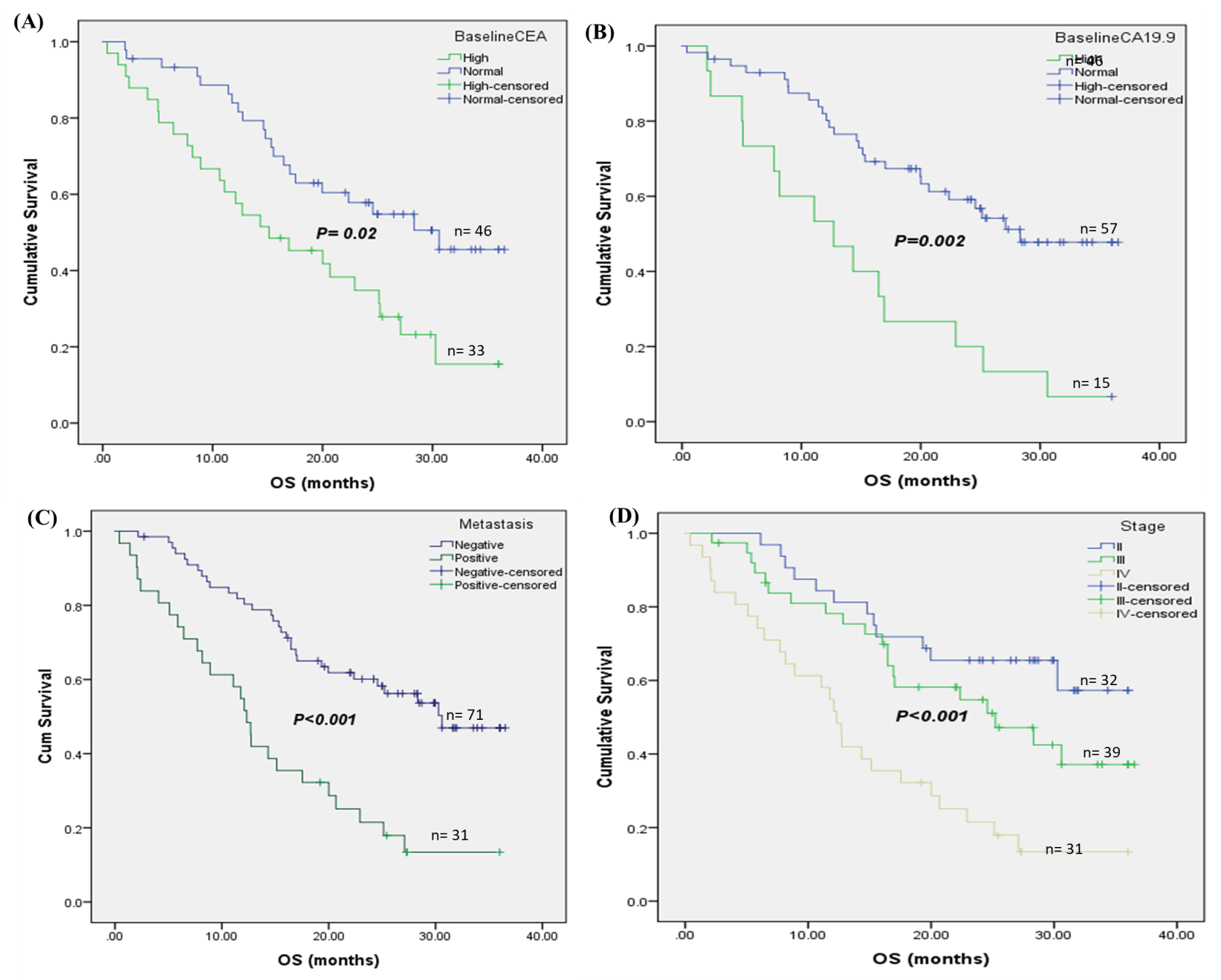


**Figure S (2):** OS of CRC patients with normal and high CEA levels (A), normal and high CA19.9 levels (B), non- metastatic and metastatic (C), and stages II, III and IV (D).


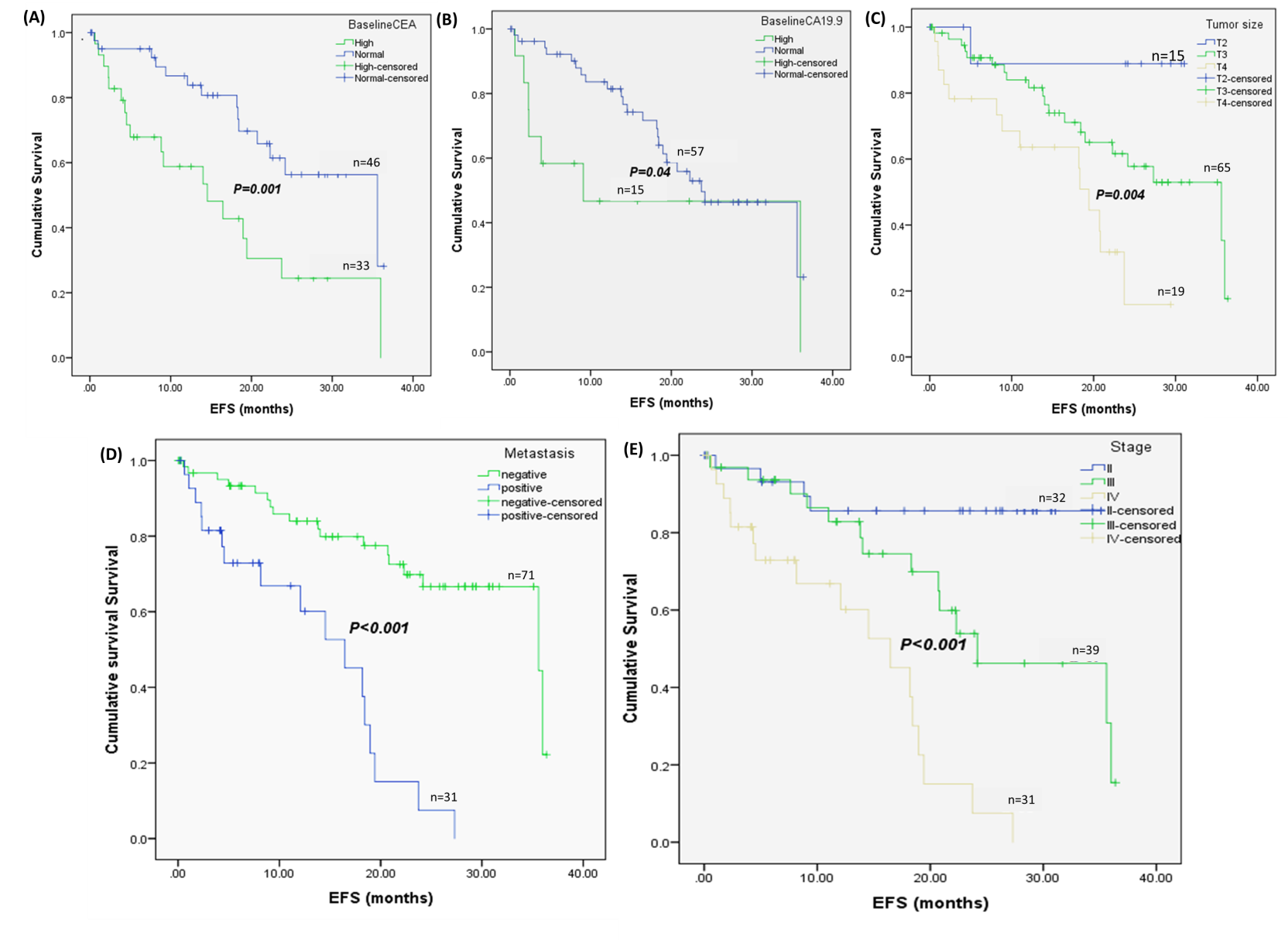


**Figure S (3):** EFS of CRC patients with normal and high CEA levels (A), normal and high CA19.9 levels (B), T2, T3 and T4 tumors (C), non- metastatic and metastatic (D), and stages II, III and IV (E)
